# Analysis on the economic effect of Sino-US trade friction from the perspective of added value

**DOI:** 10.1007/s10668-021-01390-4

**Published:** 2021-06-02

**Authors:** Zhu Zhu, Hang Zheng, Zhu Zhu

**Affiliations:** 1 Business School, Nanjing University, Nanjing, China; 2 School of Economics, Shanghai University, Shanghai, China

**Keywords:** Sino-US trade friction, Trade added value, WWZ decomposition method, Trade effect, Economic growth

## Abstract

Based on the theory of trade added value, this paper discusses the potential actual trade scale and benefit damage degree of the two countries under the background of big country game by measuring the real trade scale of China and the USA, simulating the economic impact of tariffs imposed by China and the USA and utilizing Wang–Wei–Zhu (WWZ) method to decompose the potential changes in Sino-US trade. The results show that: firstly, the size of China-US trade in terms of total value is significantly overestimated and China's overall trade with the USA in 2001–2014 was overestimated by an average of 3.06 percent, of which goods trade was overestimated by 8.06 percent. Secondly, although tariff increases can reduce the degree of trade imbalance between China and the USA to some extent, the adverse effects are mutual and global, and the European Union, the Association of Southeast Asian Nations (ASEAN), Japan and Canada become the main transfer countries of Sino-US trade. Thirdly, the pattern of China's final exports and the US' intermediate exports determines that China's trade interests are more damaged than those of the USA. It is proved that there is a big gap between China and the USA in the depth and breadth of China's participation in the value chain division of labor and the trade scale measured by Gross Domestic Product is more instructive than the total value.

## Introduction

The global value chain is undergoing profound changes, and its rapid development has become an important feature of globalization; meanwhile, the rising China has gradually become the backbone of the global value chain. As a producer and exporter of the world's major industrial products, China's gradual transition from the downstream value chain relying on foreign advanced technology and patents to the upper and middle reaches not only imperceptibly affects the adjustment of international industrial structure and the reconstruction of global value chain. It also broke the Sino-US industrial chain division of labor “balance.” The rise of China's economy has made the long-time "global hegemony" of the USA unilaterally considered threatened and provoked trade disputes. As a catalyst, the epidemic of novel coronavirus intensifies this competitive relationship, the geopolitical deterioration of China and the USA as well as the great power game results in the reconstruction of international rules (Rejeesh MR, Thejaswini P, 2020; Sundararaj V, 2019;Sundararaj V et al., 2018;Vinu S, 2016, 2019;Sundararaj, V. and Rejeesh, M.R., 2021;Tiwari, M. et al., 2021).

Sino-US trade frictions will persist in the process of realizing “Chinese Dream,” and it will become the academic circles’ key research content. Many scholars deeply analyze the causes of Sino-US trade disputes, in terms of economic motivation, the US “301” survey of China seeks to reduce the US trade deficit with China, curb the upgrading of China's industrial structure and slow down China's economic growth (Liang, 2017); Seen from the political motivation, it is to suppress China's rise, obtain the additional benefits of "US priority" and challenge the new rules of international economy and trade (Sun Li and Wang Houshuang, 2017; Yao Bo, 2018). The Sino-US trade dispute not only represents the result of Sino-US changes in the division of global value chains but also reveals the domestic contradictions in the USA. This approach could also be a means for the USA to curb China's peaceful rise in order to form the inherent inevitability of intensification of the trade friction between China and the USA and express the trend of normalization, long-term and complication (Yu Zhen et al., 2018; Li Ji and Luo Ronghua, 2019).

Among them, the serious imbalance between China and the USA caused by the inconsistency of bilateral statistical caliber is the main reason why the USA initiated a trade war. Taking 2018 as an example, according to Chinese customs statistics, the total bilateral trade in goods between China and the USA in 2018 was 633.52 billion US dollars and the surplus of goods trade was 323.333 billion US dollars. However, according to the US Department of Commerce, bilateral trade in goods totaled $659.85 billion, with a trade deficit of $419.16 billion and the difference between the two sides was $95.83 billion. Due to the inconsistent traditional trade statistics methods, the trade balance between the two countries is exaggerated; Lamy, former Director-General of the World Trade Organization (WTO), suggested that measuring trade value added could more truly reflect a country's trade level (WTO, 2011). On the basis of value added theory, Wang et al., (2017a, b) gave indicators of value chain production length, cross-border times and value chain status and Wang et al. (2017a, b) also measured the global value chain participation index based on forward and backward linkages. Huiqing and Xiaoqiang (2019) studied forward and backward linkages of global value chain and found that China's international competitiveness in the Global Value Chain (GVC) is mainly concentrated in the manufacturing sector from the low end of the value chain to the upper and middle reaches. Tan Jingrong et al. (2019) compared the manufacturing trade structure of the two countries by Wang–Wei–Zhu (WWZ) method, and it is concluded that China's exports to the USA are from low-tech manufacturing to high-end manufacturing; however, the chain of high-tech products is still the conclusion of the USA. The trade value difference between China and the USA is indeed smaller than the total value difference, and the traditional trade statistics method overestimates the trade surplus between China and the USA by 26.7%, (Chang Ran et al., 2019). There are also scholars from China and the USA as a whole trade, industry trade, labor factor income and other perspectives, decomposition by value added, who then compared total value trade with value added trade and found that US export interests suffered significantly more than China; however, the US service industry is upstream and competitive, and trade profitability is significantly stronger than that of China (Ni Hongfu, 2018; Zhang Zhiming and Du Mingwei, 2018; Cui Riming et al., 2019).

In terms of the empirical point of view, on the basis of trade value added theory, dynamic panel model is used to quantitatively analyze the influence of each GVC index on bilateral trade value added difference through measuring GVC position, forward and backward participation index. The results show that integration GVC division of labor significantly improves China's trade interests and backward participation is the main way for China to profit. American countermeasures against China significantly inhibit China's total export correlation and distinguish intermediate from final value added (Huang Yongming and Pan Anqi, 2019; Wang Lan, 2019). Another scholar used the global trade analysis model to simulate the influence of Sino-US trade friction on the global value chain activities of the two countries and even the main trading partners and found that GVC played a buffer role in Sino-US trade friction, but with the expansion of friction scale, China will be superimposed (Huang Peng et al., 2018; Shudong et al., 2019).

Therefore, based on the theory of trade added value, this paper restores the actual scale of Sino-US trade and decomposes the simulation scenario of tariffs imposed by China and the USA by WWZ method and measures potential real trade scale changes and damage between China and the USA from the perspective of global value chain. Compared with previous studies, the possible marginal contribution of this paper lies in: firstly, based on forward correlation, this paper makes a deep analysis of Sino-US trade from the perspective of added value. Point 1 is to reposition Sino-US economic and trade relations, point 2 is to focus on the balance of interests of China and the USA in the context of globalization. Secondly, some literature focuses on simulating the potential economic impact of Sino-US trade friction, but on the basis of tariff scenario simulation, this paper decomposes the potential changes between China and the USA and considering the potential real economic impact and damage scale of Sino-US trade friction, this paper aims to draw the facts and trends of Sino-US trade.

The rest is as follows: the second part is the characteristics of Sino-US trade structure from the perspective of added value, the third part is the simulation of Sino-US trade effect under the background of trade friction, the fourth part is the decomposition of simulation effect and the last part is the conclusion and implications.

## Literature review

This section presents a comprehensive literature review of the existing studies relevant to understand and analyze the objectives of the study. This section aims to discuss the characteristics of Sino-US trade structure from the perspective of added value at national level, industry level, and global value chain status’ measurement.

### Characteristics of Sino-US trade structure from the perspective of added value

#### Structural characteristics of interests at the national level

Export of value added is the main basis for measuring a country's production situation under the division of labor of global value chain (Cui Riming et al., 2019). This paper divides Sino-US bilateral trade into four parts: Domestic Value Added (DVA), Domestic Value Added Returned and absorbed by China (RDV), and Foreign Value Added (FVA) and Pure Double Counting (PDC), specific results are shown in Table 1. In terms of the total value of bilateral exports from China and the USA, the scale of bilateral trade has gradually increased and China's exports to the USA are dominated by final goods, while the US exports to China are dominated by intermediate goods.

**Table 1:** Structural characteristics of Sino-US bilateral trade interest (US $100 million, %)

China exports to USA											
	TE	TE_INT	TE_FIN	DVA	DVA _FIN	DVA_INT + rex	RDV	FVA	FVA _FIN	FVA _INT	PDC
2001	550.05	172.86	377.18	439.72	314.25	125.47	0.58	101.89	62.93	38.96	7.86
Percentage share	100.00	31.43	68.57	79.94	57.13	22.81	0.11	18.52	11.44	7.08	1.43
2014	3685.55	1581.57	2103.98	2829.64	1797.60	1032.04	16.02	596.88	306.38	290.49	243.01
Percentage share	100.00	42.91	57.09	76.78	48.77	28.00	0.43	16.20	8.31	7.88	6.59

US exports to China											
	TE	TE_INT	TE_FIN	DVA	DVA _FIN	DVA_INT + rex	RDV	FVA	FVA _FIN	FVA _INT	PDC
2001	247.15	144.70	102.46	133.74	67.82	65.91	4.09	86.22	34.63	51.59	23.10
Percentage share	100.00	58.55	41.45	54.11	27.44	26.67	1.66	34.89	14.01	20.87	9.35
2014	1489.49	978.74	510.75	933.83	383.03	550.80	24.11	391.53	127.72	263.82	140.00
Percentage share	100.00	65.71	34.29	62.69	25.72	36.98	1.62	26.29	8.57	17.71	9.40

Data source: according to RIGVC UIBE, 2016, UIBE GVC Index collation. Due to space constraints, the duplicate calculation section is not listed. ( See details http://rigvc.uibe.edu.cn/english/D_E/database_database/index.htm.)

Specifically, firstly, China's forward-linked exports to the USA are significantly higher than DVA exports, 2001–2014, China's average share of DVA exports is 79.65%, while the USA is only 56.78 percent. This is because US exports to China contain a high proportion of third-country value added and China's main exports to the USA account for a large share of the final product that causes the forward correlation American DVA proportion to be smaller than China. Secondly, America's participation in the value chain is deeper than China's, and in detail, it could be expressed as the ratio of RDV and RDV in the USA is higher than that in China, 2014, America RDV $2.411 billion, 1.62% of US exports to China, while China’s RDV is only $1.602 billion in 2014 with the proportion of 0.43. But as China's economy continues to grow, the level gap between the two parties in the value chain division of labor is gradually narrowing, 1.19 percent of the difference RDV in 2014, is 0.36% less than 1.55% in 2018. This shows that the USA is still at both ends of the "smile curve" of the value chain, China is in the middle, but is moving toward both ends. Finally, American FVA is relatively high, 26.29 percent in 2014 which is 10.09% higher than China. It not only explains why DVA account for less than China but also shows that the USA as an economic power could be better at integrating global value chain resources and the degree of participation in the division of labor is higher than that in China. Thus, it could be seen obviously that the USA is upstream of the value chain in bilateral trade while China is positioned downstream. In addition, China and the US’ bilateral trade imbalance is high.

**Fig. 1:**
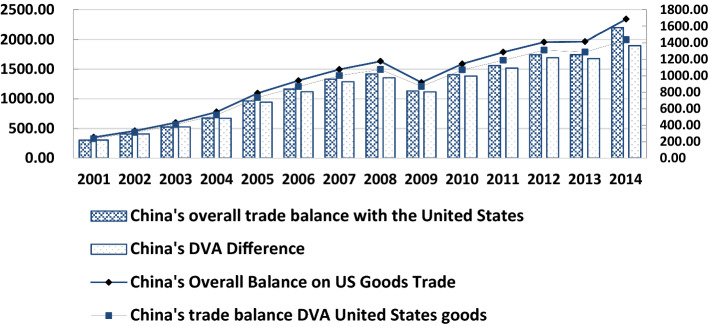
Trends in China-US bilateral trade interest balance. Data source: according to RIGVC UIBE, 2016, UIBE GVC Index collation

Figure 1 reports the trend of bilateral trade interest balance between China and the USA. From the perspective of trade interest balance, the difference in gross value is larger than that of domestic value added (DVA), which indicates that the degree of bilateral trade imbalance between China and the USA is overestimated and this trend of overestimation increases with time. Compared to domestic trade in value added, China's overall trade with the USA in 2001–2014 was on average overvalued by 3.06 percent. The average trade in goods is overvalued by 8.06. Specifically, in 2014, the value-added trade balance between China and the USA was 189.580 billion US dollars which was nearly 30 billion US dollars smaller than the total trade balance. In addition, in the trade of goods, DVA difference is $143.576 billion less than TE difference, reduced by 17.30%.

#### Characteristics of benefits structure at the industry level

Table 2 presents the structural characteristics of bilateral industrial interests between China and the USA. The table shows that the proportion of added value after decomposition changes year by year which truly reflects the degree of participation of industries in global value chain division between China and the USA. Compared to China, the USA is at both ends of value chain R & D and a sale as well as the profitability is stronger. However, with the economic construction of China's entry into WTO, the construction of free trade zone and supply-side reform, the degree of participation of various industries in the value chain division has deepened year by year and the gap with the USA has gradually narrowed.

**Table 2:** On the structure of bilateral industry interests

China exports to USA											
		TE_FIN	TE_INT	DVA	DVA_FIN	DVA_INT + rex	RDV	FVA	FVA_FIN	FVA_INT	PDC
Agriculture, forestry, animal husbandry and fisheries	2001	78.85	21.15	84.08	68.35	15.73	0.06	15.03	10.50	4.53	0.84
	2014	69.41	30.59	83.42	62.05	21.36	0.31	12.51	7.36	5.15	3.77
Mining	2001	54.25	45.75	70.71	40.22	30.49	0.13	26.84	14.03	12.80	2.32
	2014	48.90	51.10	74.15	40.38	33.76	0.53	18.14	8.52	9.63	7.18
Labor manufacturing	2001	86.03	13.97	89.76	78.82	10.94	0.04	9.74	7.22	2.52	0.47
	2014	76.74	23.26	86.85	70.44	16.41	0.20	10.19	6.31	3.88	2.76
Capital manufacturing	2001	59.66	40.34	77.35	47.43	29.92	0.14	20.73	12.23	8.50	1.77
	2014	48.86	51.14	74.38	40.09	34.29	0.52	18.12	8.77	9.35	6.98
Technology manufacturing	2001	66.32	33.68	80.52	55.13	25.39	0.15	17.79	11.19	6.60	1.54
	2014	53.25	46.75	77.42	45.65	31.77	0.53	14.95	7.60	7.35	7.10
Living services	2001	62.52	37.48	73.41	47.90	25.50	0.11	24.57	14.61	9.96	1.91
	2014	54.00	46.00	70.96	43.79	27.17	0.41	20.23	10.21	10.02	8.40
Knowledge services	2001	62.32	37.68	76.95	49.53	27.42	0.12	21.28	12.79	8.49	1.65
	2014	53.36	46.64	72.89	44.04	28.85	0.45	18.88	9.31	9.57	7.78
Public service	2001	50.05	49.95	70.89	34.85	36.04	0.11	26.93	15.20	11.73	2.07
	2014	53.74	46.26	74.46	44.71	29.75	0.46	18.09	9.04	9.06	6.99

US exports to China											
		TE_FIN	TE_INT	DVA	DVA_FIN	DVA_INT + rex	RDV	FVA	FVA_FIN	FVA_INT	PDC
Agriculture, forestry, animal husbandry and fisheries	2001	21.20	78.80	76.91	15.40	61.51	1.92	16.80	5.80	11.00	4.36
	2014	12.76	87.24	86.80	10.13	76.67	2.41	7.85	2.63	5.22	2.94
Mining	2001	25.31	74.69	53.09	14.71	38.38	2.07	35.25	10.60	24.64	9.60
	2014	14.47	85.53	48.86	7.92	40.94	1.71	30.65	6.55	24.10	18.79
Labor manufacturing	2001	34.39	65.61	65.68	25.68	40.00	2.14	25.06	8.72	16.34	7.12
	2014	38.70	61.30	77.12	33.59	43.53	1.74	14.77	5.10	9.67	6.37
Capital manufacturing	2001	36.53	63.47	56.94	23.66	33.28	1.85	32.72	12.87	19.85	8.49
	2014	28.38	71.62	55.79	20.40	35.39	1.46	28.02	7.99	20.03	14.74
Technology manufacturing	2001	48.00	52.00	60.79	35.43	25.36	1.82	29.17	12.56	16.61	8.22
	2014	47.50	52.50	65.64	39.22	26.42	1.62	23.04	8.28	14.76	9.70
Living services	2001	36.89	63.11	41.75	19.65	22.11	1.29	45.04	17.25	27.79	11.92
	2014	27.30	72.70	62.89	18.54	44.35	1.74	27.99	8.76	19.22	7.38
Knowledge services	2001	39.48	60.52	48.29	23.50	24.79	1.49	40.04	15.98	24.06	10.18
	2014	33.86	66.14	55.75	22.61	33.15	1.35	33.52	11.25	22.26	9.38
Public service	2001	36.31	63.69	50.66	21.52	29.14	1.65	37.84	14.79	23.05	9.85
	2014	32.92	67.08	58.62	22.94	35.68	1.43	30.17	9.97	20.20	9.77

*Data source* according to RIGVC UIBE, 2016, UIBE GVC Index collation. Due to space constraints, the duplicate calculation section is not listed

Firstly, China mainly exports final products and the main export industries are agriculture, forestry, animal husbandry, fishery and manufacturing. China accounted for more than 50% of DVA_FIN in both sectors in 2001–2014, while the proportion of DVA_FIN in the USA was about 50% higher during the same period. Among them, China's labor manufacturing industry has a production advantage over other industries in the “smile curve,” but in the GVC downstream position. On the contrary, the USA is in the upper GVC position with the export agriculture, forestry, animal husbandry and fishery intermediate products as the core part and its DVA_INT is 76.05 percent in 2014. This is an increase of 14.76 percent over 2001. Secondly, America's involvement in the global value chain division is broader than China's, and it could be expressed by that American FVA accounts for about twice as much as China. America's service FVA is significantly higher than China's; namely, for Chinese FVA, it comes mainly from the final products and for American FVA, it mainly comes from intermediate products. China's public service industry participates more in GVC division of labor, and the FVA in 2001_ Fin accounted for 14.88% which decreased by 6% in 2014. In the USA, life service industry is the major industry to integrate global supply resources. Compare to 2001, the FVA of the USA in 2014 is higher. Although the proportion of it has declined, it is still higher than that of China. This shows that China's participation in the value chain division is less than that of the USA. Thirdly, China's participation in the GVC division of labor is deeper than that of the USA. In terms of domestic added value’s RDV absorbed by China, although the proportion of RDV in various industries in China is increasing year by year, it is still significantly smaller than that in the USA. Among them, China's RDV share of the largest growth in the industry is mining, 2014 accounted for 0.4% higher than 2001. Capital manufacturing was the largest sector in the USA, accounting for only 1.73 percent of the RDV in 2014, but about three times that of China, and the proportion RDV agriculture, forestry, animal husbandry, fishery and living services rose up 0.48 percent and 0.45 percent, respectively, higher than China. It could indicate that the depth of China's participation in the GVC is relatively shallow and there is still a certain gap compared with the USA.

#### Global value chain status’ measurement

Table 3 compares the improved Revealed Comparative Advantage index (NRCA) 3 of China and the USA from 2000 to 2014. Among them, China has a strong comparative advantage in manufacturing, in particular, the labor manufacturing sector. It is in the upstream position of the global value chain, while the US industry is in the middle and downstream position of GVC. The average value of Normalized Revealed Comparative Advantage (NRCA) Index of China is 1.09 higher than that of the USA, so the US attack is made in China 2025, and implement long-arm jurisdiction over key "throating" technologies, so as to weaken the comparative advantage of China's manufacturing industry. The service industry has a strong comparative advantage in the USA, in which the Normalized Revealed Comparative Advantage (NRCA) Index of knowledge service industry is 1.81, while the Normalized Revealed Comparative Advantage (NRCA) Index of China's industry is only 0.44. It could indicate that the service industry of the USA is in the upstream position of GVC, while the service industry of China is in the middle and downstream position of GVC and the trade profitability is relatively weak. In addition, the Normalized Revealed Comparative Advantage (NRCA) Index of agriculture, forestry, animal husbandry and fishery in the USA is about three times that of China; therefore, there is a strong theoretical basis for China to take agricultural tax countermeasures.

**Table 3:** Global value chain position measurement in Chinese and American industries

Industry	China			USA		
	NRCA mean	Competitive advantage	GVC position	NRCA mean	Competitive advantage	GVC position
Agriculture, forestry, animal husbandry and fisheries	0.44	Comparative disadvantage	Middle and lower reaches	1.23	Medium comparative advantage	Middle
Mining	0.16	Weak comparative disadvantage	Downstream	0.16	Weak comparative disadvantage	Downstream
Labor manufacturing	1.75	Comparative advantage	Upper middle	0.66	Medium comparative disadvantage	Middle and lower reaches
Capital manufacturing	0.83	Medium comparative advantage	Middle	0.70	Medium comparative disadvantage	Middle and lower reaches
Technology manufacturing	1.24	Comparative advantage	Upper middle	1.03	Medium comparative advantage	Middle
Living Services	0.84	Medium comparative advantage	Middle	1.29	Comparative advantage	Upper middle
Knowledge services	0.44	Comparative disadvantage	Middle and lower reaches	1.81	Comparative advantage	Upper middle
Public service	0.40	Comparative disadvantage	Middle and lower reaches	1.32	Comparative advantage	Upper middle

*Data source* according to RIGVC UIBE, 2016, UIBE GVC Index collation

**Fig. 2:**
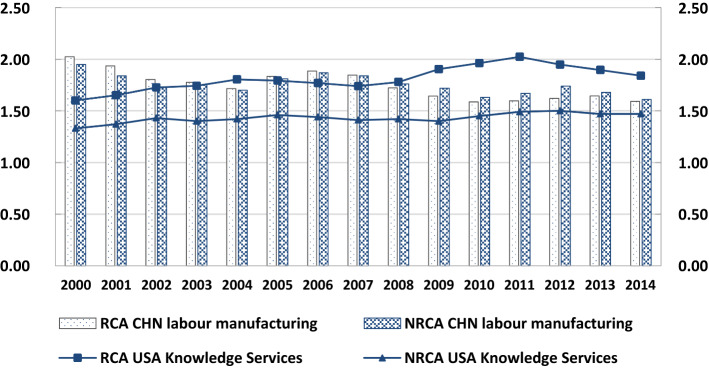
Comparative Analysis of NRCA and RCA of China-US Advantage Industries. Data source: according to RIGVC UIBE, 2016, UIBE GVC Index collation

This paper further selects China's labor manufacturing industry and the US knowledge service industry as samples and then makes a deep comparative analysis of the traditional RCA index and the value added RCA index, as shown in Fig. 2. As far as China's labor manufacturing industry is concerned, between 2000 and 2014, China's industry continues to have a strong comparative advantage which is related to China's cheap labor costs. In 2007 and before, the traditional RCA index is higher than the Normalized Revealed Comparative Advantage (NRCA) Index, and as a result, China's labor manufacturing participation GVC division of labor is overvalued, but after 2008, Normalized Revealed Comparative Advantage (NRCA) Index rose, higher than the traditional RCA index. From the perspective of knowledge-based service industry in the USA, the trend line of traditional RCA index is above Normalized Revealed Comparative Advantage (NRCA) Index, and the overall trend is flat. In 2011, the difference between the two RCA indexes is the largest, and Normalized Revealed Comparative Advantage (NRCA) Index is 0.53 lower than RCA, which indicates that the knowledge-based service industry in the USA is overvalued under the statistical caliber of gross value. Generally speaking, the traditional RCA index overestimates the comparative advantages of Chinese and American industries to a certain extent because it ignores the foreign added value, the domestic added value reversed and absorbed, and the double calculation part.

## Methodology

This section will discuss on the methods used in the current study to investigate the economic effect of Sino-US trade friction from the perspective of added value based on the mode selected for the study.

### GTAP model design

The general equilibrium model (Computable General Equilibrium, CGE), which can be used for calculation, has successfully established the quantitative relationship between the components of the economy. Compared to the input–output model, the CGE model links the sectors and industries involved in economic change. The potential economic impact of policy change is accurately simulated from macro perspective, such as trade balance, investment balance, GDP change, factor endowment, government, family, individual behavior (Dixon, 2017; Huang Peng et al., 2018; Zhengning & Xinlu, 2019). Based on this, this paper uses the GTAP model to simulate and decompose the influence of tariffs on the two countries and the world economy under the background of global value chain division of labor and to explore the real potential impact of Sino-US trade friction.

*Division of national departments* According to the GTAP 9.0 database and research content, according to the ranking of the world's largest economies, this paper combines the original 140 countries and 57 sectors into seven countries and eight industries, including China, the USA, Japan, Canada, ASEAN (except Myanmar), the European Union and other countries; industries are divided into eight categories: agriculture, forestry, animal husbandry and fishery, mining, labor manufacturing, capital manufacturing, technology manufacturing, life service, knowledge service and public service.

*Benchmark scheme setting* This paper assumes that all countries will adjust the current trade, investment, factor flow, government purchase, household consumption and other economic variables through the historical data in the Global Trade Analysis Project (GTAP) database according to the current policy before the Sino-US trade tariff is imposed.

*Scenario simulation settings* On May 5, 2019, the US government has announced a 25% tariff on $250 billion in imports. It contains $50 billion of Chinese goods announced on September 18, 2018. China then announced an increase in tariffs to 5–25% on US goods worth $60 billion. This paper, based on the US new tariff imposed on the 200 billion list of goods and China's 60 billion list of goods, set tariff shock closure conditions for benchmark scenarios.

### Simulation results

This section presents the simulation results of the study based on the GTPA model which is considered as effective tool to quantify the trade issues.

#### Macroeconomic changes in countries

Table 4 reports changes in indicators across the world relative to the baseline scenario. As two superpowers, the influence of trade friction between China and the USA is not only bilateral, but also globally. From the simulation results, it could be observed that, compared with the benchmark scenario, firstly, Sino-US trade friction caused serious damage to the two economies. China's total exports fell 5.09 percent, the USA fell 2.87 percent, total imports fell 4.61 percent, China fell 6.90 percent, and the changes in social welfare, real GDP, capital stock, household income and private consumption between China and the USA were negative. It indicates that the losses caused by trade friction were mutual and affected all aspects of social production and consumption.

**Table 4:** Variation of indicators across the world relative to baseline scenarios

Indicators	China	USA	Japan	Canada	ASEAN	EU	Other countries
Social welfare ($100 million)	– 718.11	– 407.18	70.64	57.90	42.67	125.77	260.32
Trade balance (billions of dollars)	146.84	695.51	– 176.69	– 51.35	– 47.43	– 276.39	– 290.85
GDP(%)	– 3.03	– 0.32	0.84	1.76	0.70	0.50	0.66
(%) GDP price index	– 2.59	– 0.09	0.84	1.70	0.68	0.48	0.62
GDP quantity index (%)	– 0.46	– 0.22	0.01	0.06	0.02	0.02	0.03
Terms of trade (%)	– 2.24	– 0.33	0.72	1.04	0.35	0.15	0.34
Capital stock change (%)	– 0.20	– 0.15	0.07	0.11	0.08	0.05	0.06
Household income (%)	– 1.14	– 0.30	0.14	0.37	0.23	0.08	0.14
Private consumption (%)	– 1.08	– 0.30	0.14	0.37	0.23	0.08	0.14
Changes in exports (%)	– 5.09	– 2.87	– 0.30	1.63	0.41	0.20	0.49
Changes in imports (%)	– 6.90	– 4.61	1.55	2.73	0.85	0.59	1.00

*Data source* according to the results of the GTAP simulation, the US list of Chinese goods is imposed by 25% tariff, and the Chinese US list of goods tariff rate data from the Chinese customs database

Secondly, China's economy is more damaged than the USA. China's real GDP decline is about ten times that of the USA, while China's decline is 3.03, but the USA is only 0.32; from a point of view of social welfare, China's welfare loss is 1.76 times that of the USA; and in terms of household income and private consumption indicators, China's decline is far above the USA. The reason is that China mainly exports final goods, and the trade pattern of the USA mainly exports intermediate goods determines that the American intermediate goods can quickly turn to other links in the global value chain to make up for the damage of exports.

Thirdly, the impact of trade friction is global. From the point of view of trade balance, the trade imbalance between China and the USA increased by $14.684 billion, the USA increased by $69.551 billion, and other countries, such as Japan, Canada, ASEAN, the European Union and other countries, decreased to varying degrees, with the largest reduction in the European Union, followed by Japan, which shows that trade frictions in large economies will inevitably have a deep or shallow impact on third countries and have global characteristics.

#### Changes in industry worldwide

Table 5 reports industry changes worldwide relative to baseline scenarios. On imports, the impact of tariff shock on imports in various industries in China is greater than that in the USA. Among them, China's agriculture, forestry, animal husbandry and fishery imports fell the most about five times that of the USA, the second is technology manufacturing and capital manufacturing. America's most affected imports are also manufacturing, labor, capital and technology manufacturing fell 4.98%, 4.43% and 7.61%, respectively. On exports, the export impact of various industries in the USA is greater than that of China, mainly in agriculture, forestry, animal husbandry and fishery, with a decline rate of 8.99%. Export of China's technology manufacturing industry decreased the most significantly, 9.15%, while the export of the USA was in the middle, lower than 3.75% of China's. In terms of average value, China's overall exports have a comparative advantage over the USA. The average decline rate of exports of various industries in the USA is 2.66%. The exports of the five major industries involved in trade in goods all decline. China's mining industry, labor manufacturing industry and technology manufacturing industry show a decline in exports, while other industries have increased to varying degrees, with an overall average of 1.38%. This shows that China has a comparative advantage over the USA for export, and it is also the source of Sino-US trade friction.

**Table 5:** Variation in industry worldwide relative to baseline scenario (%)

	Industry	China	USA	Japan	Canada	ASEAN	ASEAN	Other countries
Total imports	Agriculture, forestry, animal husbandry and fisheries	– 11.04	– 2.93	0.69	1.74	0.87	0.20	0.83
	Mining	– 1.04	0.51	0.00	0.63	0.13	0.30	0.23
	Labor manufacturing	– 7.29	– 4.98	1.44	2.61	0.80	0.17	0.92
	Capital manufacturing	– 9.23	– 4.43	1.66	2.67	0.72	0.14	0.98
	Technology manufacturing	– 10.10	– 7.61	3.63	3.01	1.08	0.13	1.39
	Living services	– 5.43	– 1.07	1.71	2.96	1.00	0.10	0.97
	Knowledge services	– 5.86	– 1.15	1.47	2.98	0.90	0.10	0.97
	Public service	– 6.38	– 0.70	1.52	3.32	1.33	0.10	1.20
	Average value	– 7.05	– 2.80	1.51	2.49	0.85	0.15	0.94
Total exports	Agriculture, forestry, animal husbandry and fisheries	0.28	– 8.99	0.03	– 1.36	1.34	– 0.17	0.83
	Mining	– 1.45	– 6.23	– 0.54	– 0.39	0.11	– 0.86	0.19
	Labor manufacturing	– 3.64	– 2.82	– 0.19	2.08	0.40	0.11	0.40
	Capital manufacturing	0.81	– 4.43	– 1.81	0.20	– 0.79	0.10	– 0.15
	Technology manufacturing	– 9.15	– 5.40	0.13	7.28	1.13	0.25	2.37
	Living Services	4.43	1.58	– 0.92	– 2.71	– 0.53	0.09	– 0.63
	Knowledge services	9.81	2.64	– 0.99	– 3.59	– 0.45	0.12	– 0.54
	Public service	9.94	2.33	– 1.50	– 3.92	– 1.10	0.18	– 0.93
	Average value	1.38	– 2.66	– 0.72	– 0.30	0.01	– 0.02	0.19

***Data source:*** according to the GTAP simulation results

In general, Sino-US trade friction has the greatest impact on agriculture, forestry, animal husbandry, fishery and technology manufacturing in both countries. As a developed economy, Japan is dominated by trade transfer and market segmentation between China and the USA. Its high-tech industries quickly occupy the share of the world market, and the export range has increased by 7.28%, but the exports of other industries have been negatively affected to being different.

#### Global trade transfer change

Table 6 reports changes in global trade transfers relative to the baseline scenario. China and the USA are affected by trade friction and their bilateral import and export trade turns to other countries in the world. On exports, the total exports of China and the USA decreased by US $168.888 billion and US $85.050 billion, respectively, and it could express that difference has been made compared to the bilateral imports and exports of China and the USA which could indicate that the exports of taxed industries in China and the USA turn to other countries. Reporting to the benchmark scenario, China's export transfers to Europe, ASEAN and Canada, increased by US $33.790 billion, US $14.588 billion and US $11.629 billion respectively. Similarly, European (7.188 billion US dollars), Canada (4.611 billion US dollars) and ASEAN (1.513 billion US dollars) are also the main target countries of American export trade. On imports, China's imports from other countries in the world decreased significantly, with imports from the USA decreasing by 82.713 billion US dollars (74.68%). Moreover, as the principal intermediate supplier of China's taxed industries, Japan's exports to China decreased by $7.79billion under the influence of trade friction, but increased by $12.778 billion to the USA. The USA shifted its imports to other countries except China, of which the import transfer from the European Union was US $31.253 billion, accounting for 26.04% of the export transfer market, and the market shares of Japan, Canada and ASEAN were 10.64%, 8.77% and 8.95%, respectively. Thus, the terms of trade with the EU, ASEAN, Japan and Canada rose by 0.15%, 0.35%, 0.72% and 1.04%, respectively, becoming the main target countries of Sino-US trade transfer.

**Table 6:** Variation in global trade transfers relative to baseline scenarios (billions of US dollars)

Country	China	USA	Japan	Canada	ASEAN	EU	Other countries
China	0.00	– 2208.28	– 676.29	116.29	145.88	337.90	595.62
USA	– 827.13	0.00	– 227.67	46.11	15.13	71.88	71.63
Japan	– 77.90	127.78	0.00	472.99	81.60	– 14.50	– 51.78
Canada	– 7.71	105.31	38.29	0.00	88.15	182.99	– 18.23
ASEAN	– 21.19	107.45	37.41	– 4.00	0.00	– 5.23	17,762.64
EU	– 64.51	312.53	124.50	2.79	– 5.47	0.00	– 65.10
Other countries	– 109.14	547.33	184.48	– 5.16	– 28.17	– 35.87	0.00

***Data source:*** according to the GTAP simulation results

## Discussion on economic impact simulation and decomposition of Sino-US trade friction

This section will discuss the model and the decomposition of Sino-US trade friction based on WWZ method and other decomposition methods or theories.

### Value-added decomposition theory

Koopman et al., (2014) further consummate the export decomposition model based on the theory of added value trade and forward relation, that is, KWW method, which subdivides export trade into nine parts so as to quantify a country's participation and position in global value chain. Wang et al. (2013) expand the total export decomposition model based on backward connection, that is, WWZ method, subdivide export trade into 16 parts and expand the scope of application to many levels, becoming the mainstream research ideas.

This paper draws lessons from the WWZ method, and according to the realistic basis of Sino-US trade dispute, based on the world input–output model, the total trade accounting framework in Wang et al. (2013) is extended to G countries (or regions) and each country contains *N* sectors. Table 7 shows the transnational input–output, which represents parts of the products of S countries used as intermediate inputs by R countries,$Z^{sr}$ $Y^{sr}$ means S product is used as part of the end-use product in R country,$VA^{{\text{s}}}$、$X^{{\text{s}}}$, respectively. Added value and total output represent S countries $Z$ $n \times n$ $X$ $Y$ $n \times 1$ $V$ $1 \times n$ a column vector, a row vector.

**Table 7:** Input–output table

Input–output		Intermediate use			Final requirements				Total output
		1	2	…	G	Final consumption	Capital formation	Net exports	
Intermediate inputs	1	$Z^{11}$	$Z^{12}$	…	$Z^{1g}$	$Y^{11}$	$Y^{12}$	$Y^{13}$	$X^{1}$
	2	$Z^{21}$	$Z^{22}$	…	$Z^{{2{\text{g}}}}$	$Y^{21}$	$Y^{22}$	$Y^{{2{3}}}$	$X^{2}$
	…	…	…	…	…	…	…	…	…
	G	$Z^{{{\text{g}}1}}$	$Z^{g2}$	…	$Z^{{{\text{gg}}}}$	$Y^{{{\text{g1}}}}$	$Y^{{{\text{g2}}}}$	$Y^{{{\text{g3}}}}$	$X^{{\text{g}}}$
Value added	$VA^{1}$	$VA^{2}$	…	$VA^{{\text{g}}}$					
Total inputs	$(X^{1} )^{\prime}$	$(X^{{_{2} }} )^{\prime}$	…	$(X^{{\text{g}}} )^{\prime}$					

$A^{sr} = Z^{sr} (\hat{X}^{r} )^{ - 1}$ $V^{{\text{s}}} = VA^{{\text{s}}} (X^{{\text{s}}} )^{{{ - }1}}$ $V^{r}$ $V^{{\text{t}}}$ Define the input–output coefficient, the value-added coefficient, and similar, according to the WWZ method to a country (or region) global value chain export value added decomposition, the model is as follows:

$$\begin{gathered} E^{{{\text{s}}r}} = (V^{s} B^{ss} )^{\prime}Y^{sr} + (V^{s} L^{ss} )^{\prime}(A^{sr} B^{rr} Y^{rr} ) + (V^{s} L^{ss} )^{\prime}(A^{sr} \sum\limits_{t \ne s,r}^{G} {B^{rt} Y^{tt} } ) + (V^{s} L^{ss} )^{\prime}(A^{sr} B^{rr} \sum\limits_{t \ne s,r}^{G} {Y^{rt} } ) \hfill \\ + (V^{s} L^{ss} )^{\prime}(A^{sr} \sum\limits_{t \ne s,r}^{G} {\sum\limits_{u \ne s,t}^{G} {B^{rt} Y^{tu} } } ) + (V^{s} L^{ss} )^{\prime}(A^{sr} B^{rr} Y^{rs} ) + (V^{s} L^{ss} )^{\prime}(A^{sr} \sum\limits_{t \ne s,r}^{G} {B^{rt} Y^{ts} } ) + (V^{s} L^{ss} )^{\prime}(A^{sr} B^{rs} Y^{ss} ) \hfill \\ + (V^{r} B^{rs} )^{\prime}Y^{sr} + (\sum\limits_{t \ne s,r}^{G} {V^{t} B^{ts} } )^{\prime}Y^{sr} + (V^{r} B^{rs} )^{\prime}(A^{sr} L^{rr} Y^{rr} ) + (\sum\limits_{t \ne s,r}^{G} {V^{t} B^{ts} } )^{\prime}(A^{sr} L^{rr} Y^{rr} ) + (V^{s} L^{ss} )^{\prime}(A^{sr} \sum\limits_{t \ne s}^{G} {B^{rs} Y^{st} } ) \hfill \\ + (V^{s} B^{ss} - V^{s} L^{ss} )^{\prime}(A^{sr} X^{r} ) + (V^{r} B^{rs} )^{\prime}(A^{sr} B^{rr} E^{r} ) + (\sum\limits_{t \ne s,r}^{G} {V^{t} B^{ts} } )^{\prime}(A^{sr} L^{rr} E^{r} ) \hfill \\ \end{gathered}$$

In the model,

$$\left[ {\begin{array}{*{20}c} {B^{ss} } & {B^{sr} } & \cdots & {B^{sg} } \\ {B^{rs} } & {B^{rr} } & \cdots & {B^{rg} } \\ \ldots & \ldots & \ldots & \ldots \\ {B^{gs} } & {B^{gr} } & \cdots & {B^{gg} } \\ \end{array} } \right] = \left[ {\begin{array}{*{20}c} {1 - A^{ss} } & { - A^{sr} } & \cdots & { - A^{sg} } \\ { - A^{rs} } & {1 - A^{rr} } & \cdots & { - A^{rg} } \\ \ldots & \ldots & \ldots & \ldots \\ { - A^{gs} } & { - A^{gr} } & \cdots & {1 - A^{gg} } \\ \end{array} } \right]$$ $B = (1 - A)^{{{ - }1}}$ The inverse matrix of Leontief.$L^{ss} = (1 - A^{ss} )^{ - 1}$ $L^{rr}$ $L^{{{\text{tt}}}}$ For the domestic Leontief paradox matrix, the same can be obtained. The meaning of each part of the total export value decomposition model is shown in Table 8.

**Table 8:** WWZ breakdown of composition and meaning

Symbol	Number of items	Meaning	
	TE	1–16	Total exports
DVA	DVA_FIN	1	Domestic value added for final exports
	DVA_INT	2	Intermediate exports absorbed by direct importing countries
	DVA_INTREX	3–5	Intermediate exports absorbed by direct importing country production to third countries
	RDV	6–8	Domestic value added returned and absorbed by the country
FVA	MVA	9、11	Value added implied by going abroad
	OVA	10、12	Export implied third-country value added
	FVA_FIN	9–10	Foreign value added for final exports
	FVA_INT	11–12	Foreign value added for intermediate exports
PDC	DDC	13–14	Pure double counting of domestic accounts
	FDC	15–16	Pure double counting of foreign accounts

### Decomposition of changes in Sino-US trade interests

According to the origin and final absorption destination of trade products, the total value after the change is decomposed into 16 items by WWZ method and finally classified into ten items. Table 9 reports changes in Sino-US trade interests relative to the benchmark scenario.

**Table 9:** Decomposition of changes in Sino-US trade interests relative to the benchmark scenario ($100 million)

Change value (Billions of US dollars)		TE	TE_INT	TE_FIN	DVA	DVA_FIN	DVA_INT + REX	RDV	FVA	FVA_FIN	FVA_INT
China	Total exports	– 1688.88	– 174.09	– 1514.8	– 1136.97	– 252.13	– 884.83	573.75	– 10,991.1	– 1262.66	– 9728.44
	Total imports	– 1107.58	– 848.82	– 258.78	– 623.54	– 201.9	– 421.63	– 2640.94	– 165.92	– 56.87	– 109.05
	Trade balance	– 581.3	674.73	– 1256.02	– 513.43	– 50.23	– 463.2	3214.69	– 10,825.18	– 1205.79	– 9619.39
USA	Total exports	– 850.05	– 493.57	– 356.48	– 757.52	– 393.97	– 363.55	– 2594.24	– 52.88	37.48	– 90.36
	Total imports	– 1007.9	– 422.3	– 585.6	– 1751.07	– 37.17	– 1713.89	788.21	– 8813.47	– 548.44	– 8265.03
	Trade balance	157.85	– 71.27	229.12	993.55	– 356.8	1350.34	– 3382.45	8760.59	585.92	8174.67
China and the USA	China Exporting America	– 2208.28	– 1302.61	– 905.67	– 1567.35	– 267.55	– 1299.8	726.2	– 10,423.06	– 638.12	– 9784.94
	US Export	– 827.13	– 673.93	– 153.21	– 422.81	– 125.44	– 297.36	– 2627.38	– 117.91	– 27.76	– 90.15
	Trade balance	– 1381.15	– 628.69	– 752.46	– 1144.54	– 142.11	– 1002.44	3353.58	– 10,305.15	– 610.36	– 9694.79

***Data source:*** according to the GTAP simulation results, using R software decomposition

^2^Due to space constraints, the duplicate calculation section is not listed. Among them,TE_FIN = DVA_FIN + FVA_FIN;FVA = FVA_INT + FVA_FIN;TE_INT = DVA_INT + DVA_INTREX + RDV + FVA_INT + PDC;DVA = DVA_INT + DVA_FIN + DVA_INTREX

From the simulation results of decomposition, China's trade structure mainly exports final goods and imports intermediate goods, while the US exports intermediate goods and imports final goods. But the global characteristics of trade friction make China and the US trade interests damaged, and China's negative impact is greater than the USA. Among them, trade between China and the USA measured on total export value overestimates trade losses, namely China's export DVA fell by $113.697 billion, accounting for 67.32 per cent of the decline in total exports, TE overvalued $55.191 billion, and US export DVA fell by $75.752 billion, TE overvalued $9.253 billion. The difference of overvalued scale depends on the difference of bilateral trade structure between China and the USA. As far as the trade balance is concerned, China's import and export trade balance has actually decreased by US $51.343 billion, which is 11.68% overestimated in terms of gross value. However, the change of US import and export trade balance has been underestimated by US $83.570 billion, which shows that under the influence of tariffs, in order to satisfy domestic needs, the US imports transfer faster than China.

Affected by the trade structure, China's domestic value added exports to the USA fell by $156.735 billion, DVA exports to China fell by $42.281 billion. Although China's export DVA to the USA has decreased more, some of the RDV returned to China from the USA and were absorbed increased by $76.22 billion. This suggests that China indirectly exports more products to the USA through third countries which is consistent with Xiao Zhimin's (2019) study. Similarly, the Sino-US trade surplus fell by $138.15 billion under tariff pressure, DVA reduction of $114.454 billion, changes in trade imbalances were overestimated by 17.13 per cent (Table 5). Therefore, under the statistics of total export value, the change degree of Sino-US trade imbalance is overestimated which is also the more realistic forward-looking point of view of the world trade organization to measure trade gains from the perspective of value added.

### Decomposition of Sino-US trade sector

Table 10 reports the decomposition of changes in Sino-US trade interests relative to the benchmark scenario. Total export statistics show a large fluctuation in the reduction of exports from various industries in China, of which the total export of China's technology manufacturing industry has declined the most, with DVA damage of US $48.552 billion, and the largest increase is in the domestic service industry, with DVA benefits of US $1.062 billion. This is due to the large differences in value chain status and the uneven distribution of superior and inferior industries. According to the total value statistics, the average export damage of various industries in the USA is US $10.626 billion, which is 0.94% overestimated compared with the average damage scale of DVA of US $9.469 billion.

**Table 10:** Decomposition of Sino-US trade interests relative to benchmark scenario ($100 million)

China's export change decomposition											
		TE	TE – FIN	TE – INT	DVA	DVA _FIN	DVA _INT + rex	RDV	FVA	FVA_FIN	FVA _INT
Agriculture, forestry, animal husbandry and fisheries	USA	– 4.83	– 0.01	– 4.82	– 3.22	0.00	– 3.22	2.27	– 51.38	– 0.02	– 51.36
	ROW	1.63	– 1.85	3.48	– 1.63	0.35	– 1.98	1.97	– 53.48	– 2.21	– 51.27
Mining	USA	– 6.1	0.00	– 6.10	– 13.53	0.00	– 13.53	12.37	12.45	0.00	12.45
	ROW	– 1.53	0.00	– 1.53	– 11.86	0.00	– 11.86	11.89	12.44	0.00	12.44
Labor manufacturing	USA	– 403.29	– 9.53	– 393.75	– 355.89	0.67	– 356.56	236.88	– 3554.99	– 10.20	– 3544.78
	ROW	– 286.02	– 260.67	– 25.35	– 250.21	18.30	– 268.51	208.04	– 3817.62	– 278.97	– 3538.65
Capital manufacturing	USA	– 169.55	– 3.00	– 166.55	– 226.04	– 0.26	– 225.78	145.14	– 1727.25	– 2.74	– 1724.51
	ROW	25.38	– 8.91	34.30	– 165.73	– 0.78	– 164.95	117.44	– 1726.10	– 8.13	– 1717.97
Technology manufacturing	USA	– 1315.13	– 574.59	– 740.55	– 722.78	– 13.63	– 709.16	329.22	– 5038.76	– 560.96	– 4477.80
	ROW	– 1178.27	– 934.86	– 243.40	– 485.52	– 22.17	– 463.35	239.65	– 5346.96	– 912.69	– 4434.26
Living services	USA	– 2.79	– 5.97	3.18	– 1.08	– 3.52	2.44	0.45	– 2.76	– 2.45	– 0.30
	ROW	29.28	0.92	28.36	10.62	0.54	10.08	– 2.07	0.14	0.38	– 0.24
Knowledge services	USA	– 21.47	– 26.01	4.54	– 15.62	– 20.01	4.38	0	– 5.06	– 6.01	0.94
	ROW	2.91	– 23.45	26.36	– 6.42	– 18.03	11.61	– 2.18	– 4.41	– 5.41	1.00
Public service	USA	– 285.11	– 286.55	1.43	– 229.19	– 230.81	1.62	– 0.13	– 55.31	– 55.74	0.43
	ROW	– 282.27	– 285.97	3.70	– 226.22	– 230.34	4.13	– 0.98	– 55.11	– 55.63	0.52

Decomposition of US export change											
		TE	TE – FIN	TE – INT	DVA	DVA _FIN	DVA _INT + rex	RDV	FVA	FVA_FIN	FVA _INT
Agriculture, forestry, animal husbandry and fisheries	CHN	– 107.76	– 7.71	– 100.05	– 8.04	– 7.17	– 0.88	– 0.45	– 5.09	– 0.54	– 4.55
	ROW	– 78.54	– 4.41	– 74.14	– 4.68	– 4.10	– 0.58	– 0.49	– 4.84	– 0.31	– 4.54
Mining	CHN	– 30.57	– 0.04	– 30.52	88.13	– 0.03	88.17	– 72.80	– 3.24	– 0.01	– 3.23
	ROW	– 25.63	– 0.03	– 25.60	79.00	– 0.03	79.02	– 75.67	– 3.24	– 0.01	– 3.23
Labor manufacturing	CHN	– 238.32	– 22.10	– 216.23	– 233.56	– 10.32	– 223.24	– 627.39	– 79.54	– 11.78	– 67.76
	ROW	– 82.98	75.95	– 158.93	– 176.27	35.47	– 211.74	– 623.65	– 27.22	40.48	– 67.71
Capital manufacturing	CHN	– 78.97	– 0.75	– 78.21	52.25	– 0.69	52.95	– 319.64	– 3.32	– 0.06	– 3.26
	ROW	– 52.96	2.85	– 55.81	7.07	2.62	4.45	– 301.89	– 3.01	0.24	– 3.25
Technology manufacturing	CHN	– 367.99	– 121.09	– 246.90	– 322.79	– 105.76	– 217.03	– 1609.3	– 26.63	– 15.33	– 11.30
	ROW	– 201.41	40.86	– 242.26	– 174.87	35.68	– 210.55	– 1588.4	– 6.43	5.17	– 11.61
Living services	CHN	– 0.96	– 0.37	– 0.59	0.08	– 0.34	0.43	0.41	– 0.05	– 0.03	– 0.03
	ROW	– 72.64	– 85.86	13.22	– 82.09	– 79.94	– 2.15	– 0.12	– 5.94	– 5.92	– 0.02
Knowledge services	CHN	– 1.98	– 0.70	– 1.28	1.50	– 0.68	2.18	1.63	– 0.04	– 0.02	– 0.02
	ROW	– 61.22	– 106.07	44.84	– 123.24	– 103.21	– 20.03	– 3.12	– 2.87	– 2.86	– 0.01
Public service	CHN	– 0.59	– 0.45	– 0.14	– 0.38	– 0.45	0.07	0.16	0.00	0.00	0.00
	ROW	– 274.66	– 279.77	5.11	– 282.43	– 280.45	– 1.98	– 0.91	0.68	0.68	0.00

***Data source:*** according to the GTAP simulation results, using R software decomposition. Due to space constraints, the duplicate calculation section is not listed. USA, ROW is the destination of Chinese exports; CHN, ROW is the destination of American exports

In bilateral trade statistics between China and the USA, the impact of tariff impact on China's exports is higher than that of the USA. According to the average statistics, the average scale of damage to China's exports to the USA is US $19.592 billion, and the total value is overestimated by 29.02%; the average loss of US exports to China is 10.339 billion US dollars, 51.11% over the value added statistics. But manufacturing, especially technology and labor, is among the hardest hit, with mining and services doing little.

Manufacturing is a key target industry in China's taxable list, so China's export trade to the USA is most affected by tariff shocks. Among them, the technology manufacturing industry suffered the most serious damage. In terms of the total value of exports, China's exports to the USA decreased by 131.513 billion US dollars, while in terms of value added, the decline was 72.278 billion US dollars, which was overestimated by 45.04%. This is because the industry's exports to the USA implied a large amount of foreign added value, which made it overestimated. The labor manufacturing industry ranked second in the damage degree. China's exports to the USA decreased by 35.589 billion US dollars, accounting for 88.25% of the total export value. The damage degree of China's exports to the USA in this industry was overestimated by 11.75%. In addition, the public service industry exported from China to the USA was the hardest hit, and the real export scale decreased by US $22.919 billion.

In contrast, the USA has an obvious comparative advantage in the export of agricultural products and manufacturing industries to China and China is also the largest trade partner of the USA. Thus, agricultural products and manufacturing industries of the USA are the industries that China vigorously counterattacks taxation. Thus, the scale of the export of these two kinds of industries to China is correspondingly reduced. The biggest decline was in technology-manufacturing exports to China, where the real scale was damaged by $32.279 billion and the damage was overestimated by 12.28 percent. The second is the labor manufacturing industry, with a total value of $23.832 billion, an overestimation of 1.99% compared with the added value. Agriculture, forestry, animal husbandry and fishery ranked the third in the reduction. As more than half of the agricultural products of the USA are exported to China, China's imports from the USA have decreased significantly after being affected by tariffs. As China's tax list mainly involves goods, the reduction of various service industries exported to China by the USA is not significant.

## Conclusions and implications

Based on the theory of value-added trade, this paper measures the actual scale of Sino-US trade, uses GTAP to simulate the situation of Sino-US bilateral tariffs and utilizes WWZ decomposition framework to decompose the changes of Sino-US bilateral trade, bilateral trade and industry. By decomposing the changes of China and the USA, bilateral trade and industry, this paper not only deeply analyzes the real scale of China-US trade, but also makes a detailed analysis of the potential trade scale changes and damage of the two countries and the world under the background of China US trade friction. According to the results of the study, the following conclusions can be drawn: firstly, the scale of Sino-US trade in terms of total value is overestimated, and the overall trade between China and the USA is overestimated by 3.06% on average in 2001–2014, of which the average trade in goods is overestimated by 8.06% on average. Taking the Normalized Revealed Comparative Advantage (NRCA) Index as an example, the average Normalized Revealed Comparative Advantage (NRCA) Index of the US knowledge-based services industry in 2001–2014 was 1.43; compared to 1.81 under the traditional caliber, it was overestimated by 0.38. Secondly, although tariffs can reduce the degree of trade imbalance between China and the USA to some extent, the adverse effects are mutual and global. Compared to the tariff increase, China's total exports fell 5.09, total imports fell 6.90, and total imports and exports of the USA fell 2.87 percent and 4.61 percent, respectively. And the European Union, ASEAN, Japan and Canada become the main transfer countries of Sino-US trade. Thirdly, the differences in bilateral trade structure between China and the USA determine that China's trade interests are more damaged than those of the USA. China mainly exports final products, and labor manufacturing industry participates in the division of labor in the global value chain, which has the advantage of production in “smile curve.” The USA mainly exports intermediate products, in the value chain R & D and sales at both ends, profitability is stronger. Under total value statistics, China's exports to the USA suffered an average of $27.602 billion which was overvalued by 29.02% compared with added value, while the US exports to China suffered an average of $10.339 billion, overvalued by 51.11%, with technology manufacturing among the top industries. However, as China gradually changes from a manufacturing power to a manufacturing power, the gap between China and the USA is gradually narrowing, whatever it is the scale of trade or GVC status. According to the results of the study, the following conclusions can be drawn. Firstly, the scale of Sino-US trade in terms of total value is overestimated, and the overall trade between China and the USA is overestimated by 3.06% on average in 2001–2014, of which the average trade in goods is overestimated by 8.06% on average.

### Managerial implications

As the core competitiveness of a country, high-tech is not only the "powerful factor" for China to enhance its international competitiveness and enhance its international voice, but also the focus of the USA. Since the Sino-US trade war in 2018 to novel coronavirus epidemic now, the implementation of China's "powerful factor" technology long-arm jurisdiction, high-tech has been the core focus of the USA to suppress China. Facing the trade friction between China and the USA in the post-epidemic era, firstly, China needs to continue to develop high-tech manufacturing industry, tilt the policy of core technology manufacturing industry, increase R & D investment in scientific research institutes, universities and other related specialties, and encourage more outstanding talents to invest in industries such as chips and 5G. Secondly, enhancing regional cooperation and making full utilization of the 19 FTAs signed with 26 countries or regions, especially the newly signed RCEP, to integrate Asia–Pacific resources and promote regional cooperation can not only buffer the losses caused by Sino-US trade frictions, but also enhance regional partnerships. Lastly, China should speed up the FTA negotiations between China, Japan and South Korea, the China–EU investment agreement negotiations and the upgrading of existing FTA, expand China's trade circle in breadth and depth and combine the "Belt and Road" initiative to actively regulate the high-standard and high-level free trade area network system to enhance the international voice.
